# Transitions in Smoking Behaviour and the Design of Cessation Schemes

**DOI:** 10.1371/journal.pone.0047139

**Published:** 2012-10-11

**Authors:** Johan Grasman, Raoul P. P. P. Grasman, Han L. J. van der Maas

**Affiliations:** 1 Biometris, Wageningen University and Research Centre, Wageningen, The Netherlands; 2 Department of Psychology, University of Amsterdam, Amsterdam, The Netherlands; The University of Auckland, New Zealand

## Abstract

The intake of nicotine by smoking cigarettes is modelled by a dynamical system of differential equations. The variables are the internal level of nicotine and the level of craving. The model is based on the dynamics of neural receptors and the way they enhance craving. Lighting of a cigarette is parametrised by a time-dependent Poisson process. The nicotine intake rate is assumed to be proportional with the parameter of this stochastic process. The effect of craving is damped by a control mechanism in which awareness of the risks of smoking and societal measures play a role. Fluctuations in this damping may cause transitions from smoking to non-smoking and vice versa. With the use of Monte Carlo simulation the effect of abrupt and gradual cessation therapies are evaluated. Combination of the two in a mixed scheme yields a therapy with a duration that can be set at wish.

## Introduction

Nicotine addiction is a significant worldwide health problem. We present a dynamical model that focuses on three key variables that play a role in the development and persistence of smoking addiction. The purpose is not to model each and every (neurobiological and psychological) detail of the processes that underlie the addiction, but to abstract away from low-level dynamics to the dynamics of these three summary variables that capture some of the most important aspects of smoking addiction. The advantage of doing so lies in that we provide a level of explanation of prominent phenomena observed in nicotine cessation research. This approach promotes the understanding of transitions in smoking behaviour in terms of some well understood mechanisms from mathematical bifurcation theory applied to the attractor dynamics of a nonlinear system.

Nicotine addiction arises from the dynamics of specific receptors on the membrane of neurons in the brain [1]. When these so-called nicotinic acetylcholine receptors (NARs) are turned on, dopamine activity is enhanced leading in turn to a need of keeping nicotine in the body at a high level. We see this need as the basis of craving. Its action is strongly felt during a period of nicotine withdrawal [2]. Opposing effects may come from e.g. the understanding that smoking is harmful. Next to this form of control there is also the effect of societal measures that may forbid smoking in certain areas. The understanding of harmful effects in the individual may be formed by information that is provided by society [3] and may be reinforced by peer pressure. Peers may as well stimulate the person to continue or (re)start smoking (negative control).

Based on the above description of the driving mechanism, that makes people continue to smoke, we formulate the elements that will constitute a dynamical model of the state of an individual with respect to the smoking habit. We identify the state of a person by two variables: *N* for the amount of nicotine in the body and *C* for the level of craving. Self (and societal) control *S*, that may lower this level, we take as an external variable in the process of interaction between nicotine intake and craving. This control may come from the conscience of the smoker himself in the form of self-efficacy [4] or from other persons influencing the behaviour of the person. In line with the above coupling of craving and self-control, we use for these two quantities the same scale.

Craving is a mental state resulting from neural processes that can be seen as dopamine-gated learning [5]. Since the number of NARs is limited, we expect that the turn on rate of NARs will depend on the number of turned off NARs and the amount of nicotine in the body. We also expect that the state of craving *C* is steered by the action of the set of NARs: there will be a maximum value for *C* that is reached when all receptors are turned on. We set this maximum value equal to 1. Furthermore, we assume that the craving dynamics can be modelled as a simple second order chemical reaction meaning that the increase of *C* per unit of time is proportional to the product (1 - *C*)*N*. Both the nicotine amount in the body and the level of craving decay in time if no nicotine is taken up; the decay of nicotine in the body is in the order of hours and that of craving in the order of months. We deal with the day-night rhythm by skipping the night and replacing the 24 hours of a full day by 16 hours.

**Figure 1:**
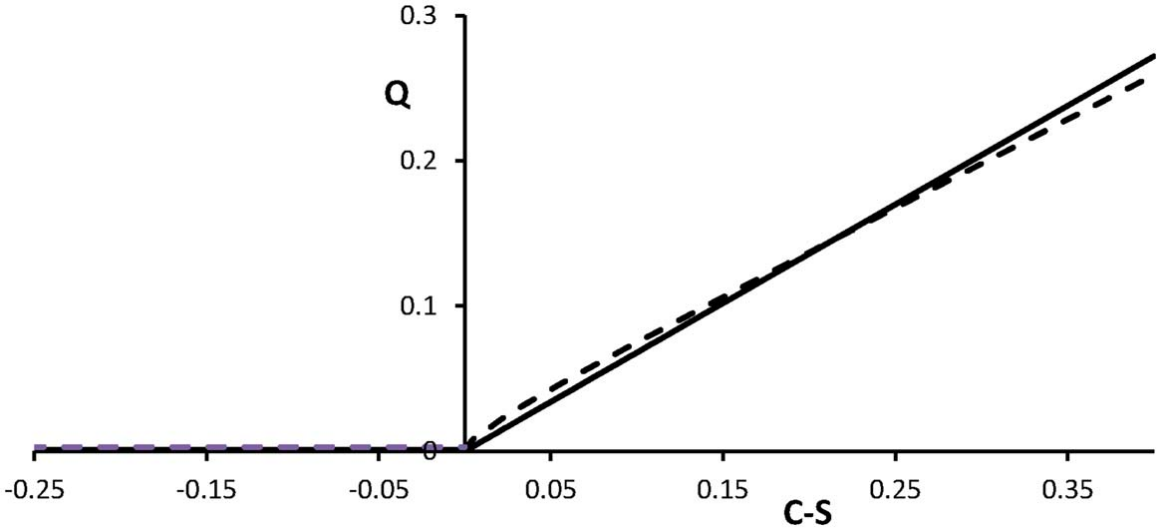
Nicotine intake *Q* as a function of the craving *C* and control *S*. Assuming a fixed urge to light a cigarette (solid) with λ = 0.68 (*C* – *S*) and the case of an urge increasing linearly in time (dashed) with λ(*t*) = (0.6+0.013*t*) (*C* – *S*).

## Materials and Methods

The intake rate of nicotine is a function of *C* – *S,* being craving corrected by self-control. We model the intake of nicotine as follows. The event of lighting a cigarette is assumed to be a Poisson process with intensity parameter λ, see Appendix. This means that per unit of time an average of λ cigarettes are smoked and that the time between the lighting of two cigarettes is exponentially distributed with the same parameter λ. The quantity *C* – *S* depends upon this parameter λ. Let this be a linear relation. The intake rate of nicotine, indicated by *Q* [mg/hr], will therefore also be a linear function of *C* – *S* when this expression takes a positive value; for negative values it will be set zero, see Figure 1. From the literature [2] it is known that, if lighting of a new cigarette is postponed, the urge may increase. In [6] three stages in this urge of lighting a cigarette are discerned. We model this progression in a continuous way by making the Poisson parameter λ time dependent. Assuming a linear dependence upon time and adding a multiplicative factor from control corrected craving we obtain.

**Figure 2:**
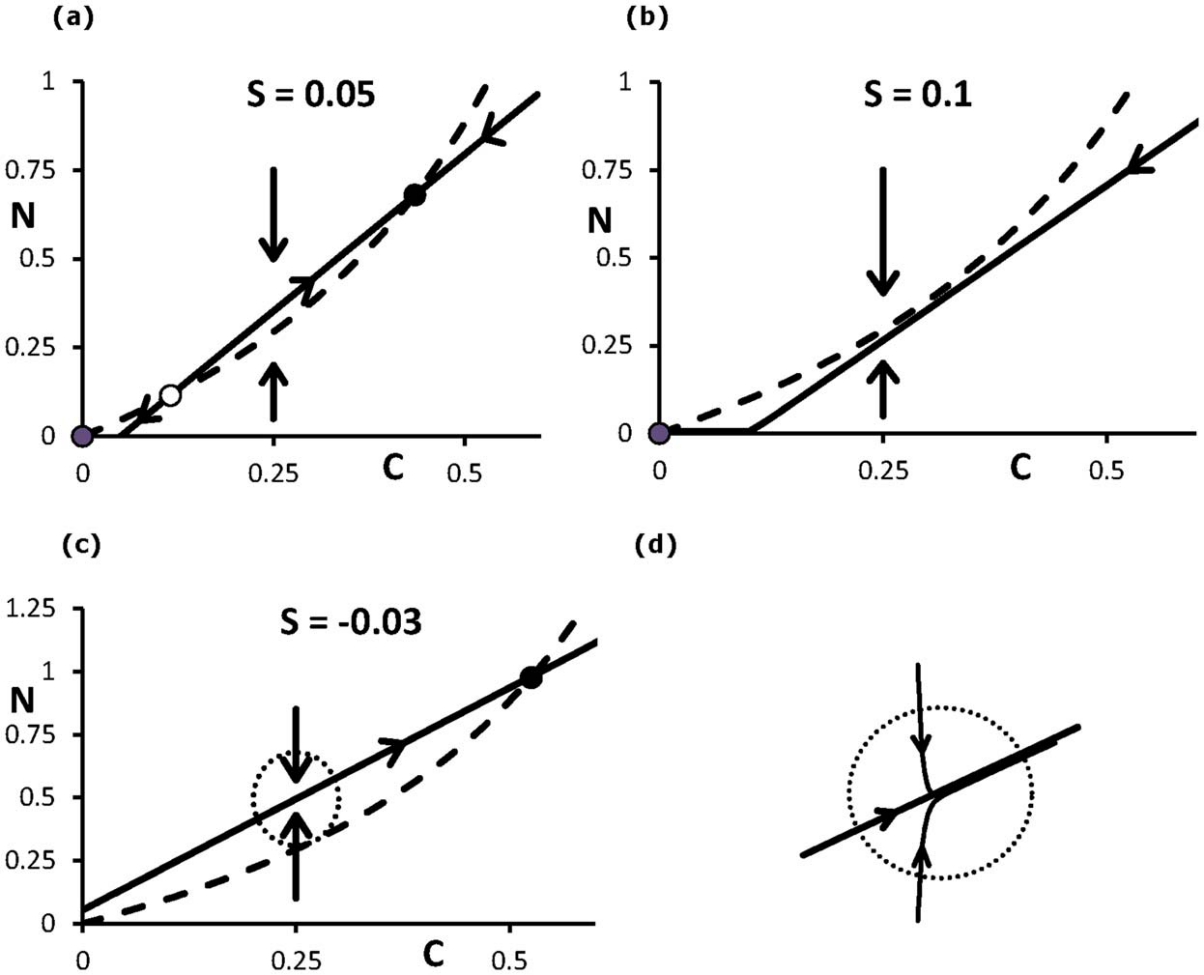
The dynamics of the system (1ab). The equilibria are at the intersection of the two nullclines, *dN/dt* = 0 (solid) and *dC/dt = *0 (dashed). If starting in an arbitrary point of the plane the system rapidly moves to a quasi-steady state near the nullcline *dN/dt* = 0. Along this curve it goes next more slowly in the direction of a stable equilibrium: (a) For a control *S* = 0.05 there are two stable equilibria (•) of respectively steady smoking and no smoking. (b) For *S* = 0.1 only the (stable) no-smoking equilibrium is left. (c) For *S* = −0.03 only the smoking equilibrium is left as stable limit solution. (d) Blow up of approach of quasi-steady state, see Figure 2c.

**Figure 3:**
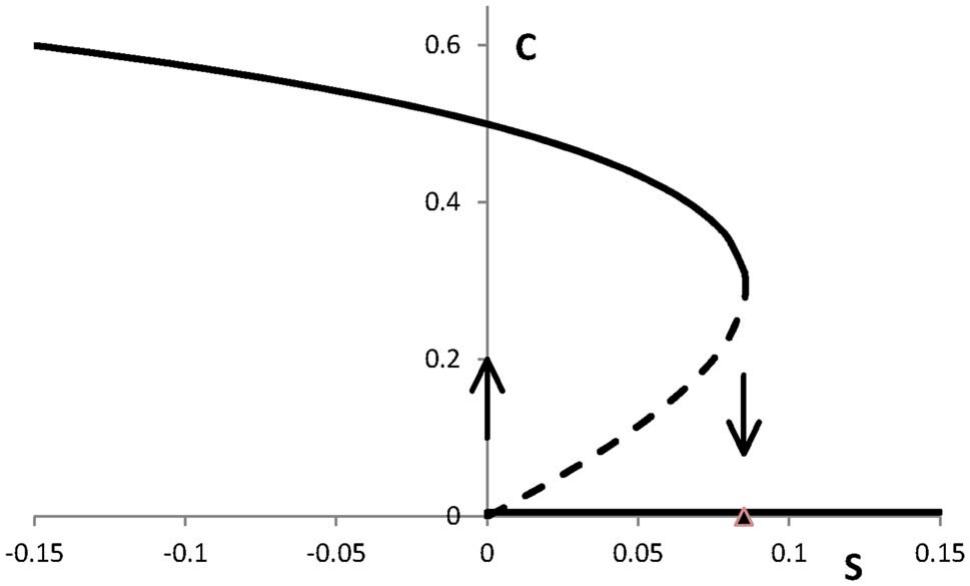
Diagram of the stable (solid) and unstable (dashed) equilibrium states of the system (1ab) as a function of *S* with the values of the other parameters given in the text. If *S* varies the system exhibits hysteresis: depending on the history of *S* the system is in one of the two stable states arising in the interval [0, 0.085].



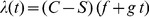
(1)with the last cigarette finished at *t* = 0. The moment *T* when a next cigarette is lighted has an expected value that can be computed numerically from formula (A2) in the Appendix S1. Since we neglect the time of smoking a cigarette, the reciprocal will be a measure for the nicotine intake. In Figure 1 we compare the nicotine intake *Q* as a function of *C* – *S* for the case of a constant urge (*g* = 0) with an example driven by an increasing urge (*g* >0), see (1). At the end of this section we will make it plausible that from now we may restrict our analysis to the case *g* = 0.

**Figure 4:**
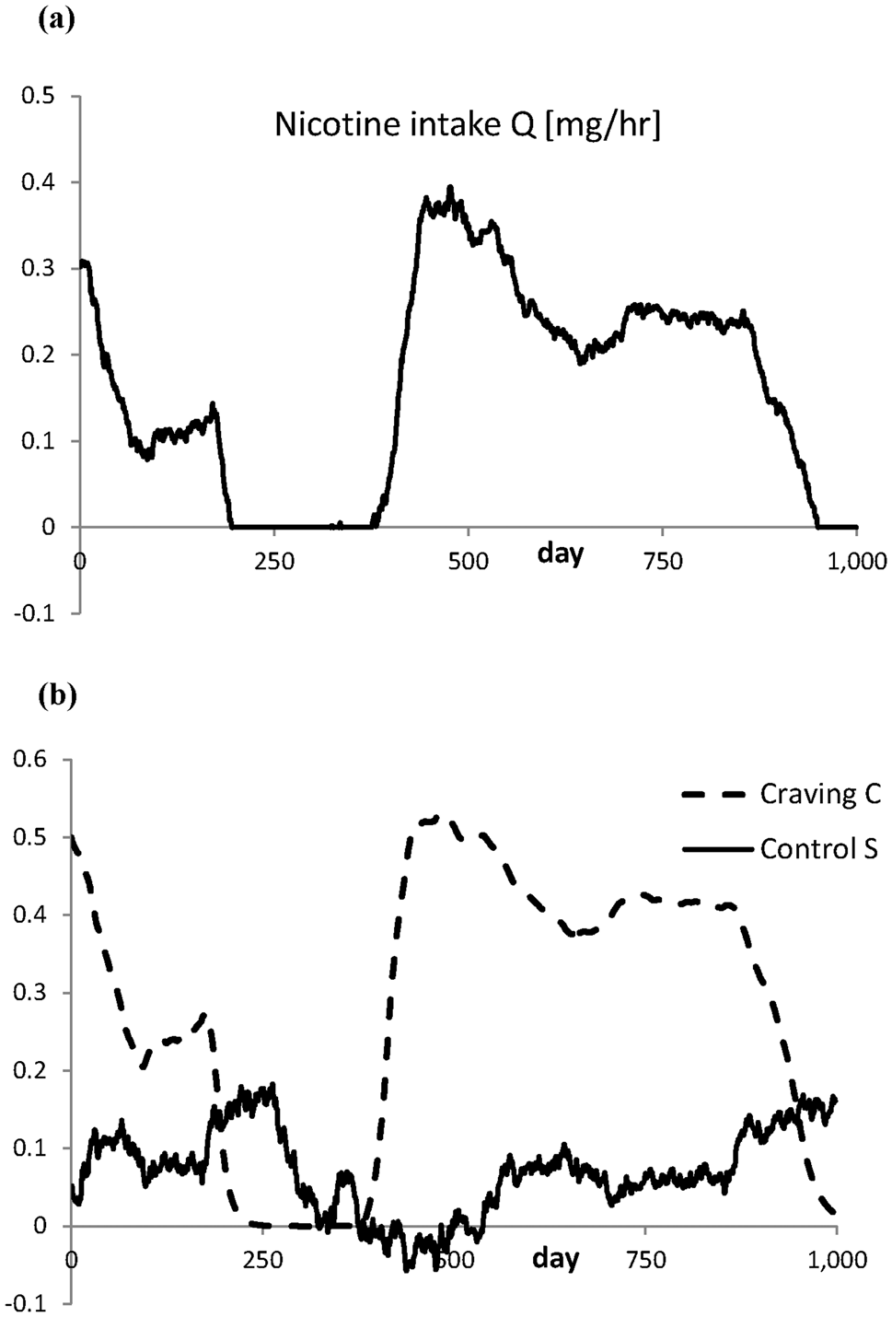
One realization of the dynamics of the system (3) with *k* = 75 and *S* = 0.05 over an interval of 1000 days. (a) The intake *Q* [mg/hr] as given by the first term of the right hand side of Eq.(3a). (b) The craving intensity *C* and the control *S*. Note that a relapse takes place when *S* switches to negative values.

**Figure 5:**
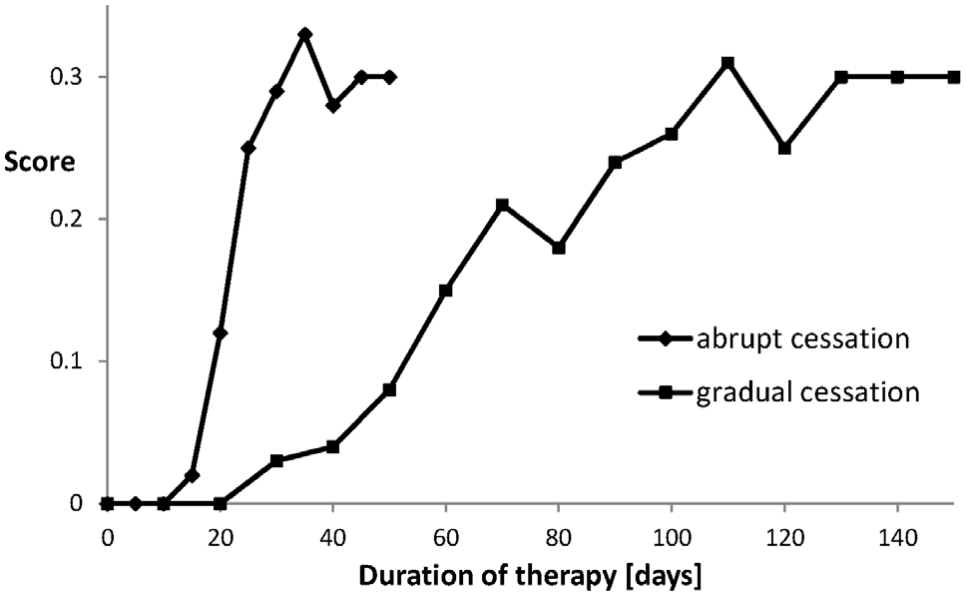
Scores for two types of cessation strategies as described in the text. The vertical axis give the fraction of successes out of 100 realisations. Success means that during one year after ending the therapy the person has a nicotine intake that stays below 5% of the intake before the therapy.

The variables *N* (amount of nicotine in body) and *C* (craving intensity) constitute the state variables of a dynamical system; their change is defined by the following set of differential equations in which [·]^+^ denotes that 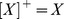 for 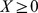 and 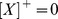 for 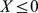:
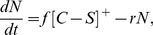
(2a)

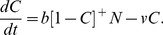
(2b)


The values of the rate coefficients *f*, *r*, *b* and *v* are derived as follows. Note that the value of the decay parameter *r* corresponds with a half time of 1.8 [hr] found in the literature [7]: *r* = ln(2)/1.8 = 0.385 [1/hr]. The value of the other decay parameter *v* matches the outcome of a study [8] where the urge for a cigarette from craving is registered ultimately after 26 days of no smoking in a group of 214 persons. Assuming that at day 26 the level of craving is 1/10 of its original value we obtain *v* = ln(10)/(26x16) = 5.54×10^−3^[1/hr]. Eq.(2a) describes the change of the nicotine amount in the body and Eq.(2b) that of the craving intensity. The values of the parameters *f* and *b* and the control parameter *S* will differ from person to person; we make an estimate of these parameters for a moderate smoker with a mean of λ = 0.8 cigarette per hour (12.8 cigarettes a day) who is not exposed to any positive or negative control: *S* = 0. The variable *C* takes values on the interval [0, 1] and scales with the fraction of turned on NARs. We take for such a smoker an equilibrium state *C* = 0.5. Assuming that one cigarette contains 1.7 [mg] nicotine [9] and only 25% is taken up in the body [10] the person has an average nicotine intake of *Q* = 0.8×1.7×0.25 = 0.340[mg/hr], so that *f* = 0.340/0.5 = 0.680[mg/h]. In the equilibrium *N* = *v*/*b,* see (2b), while from (2a) follows that also *N* = 0.5*f*/*r,* so that *b* = 2*rv*/*f* = 6.27×10^−3^[1/(mgxh)]. Thus, we have

*f* = 0.680, *r* = 0.385, *b* = 6.27×10^−3^ and *v* = 5.54×10^−3^.

**Figure 6:**
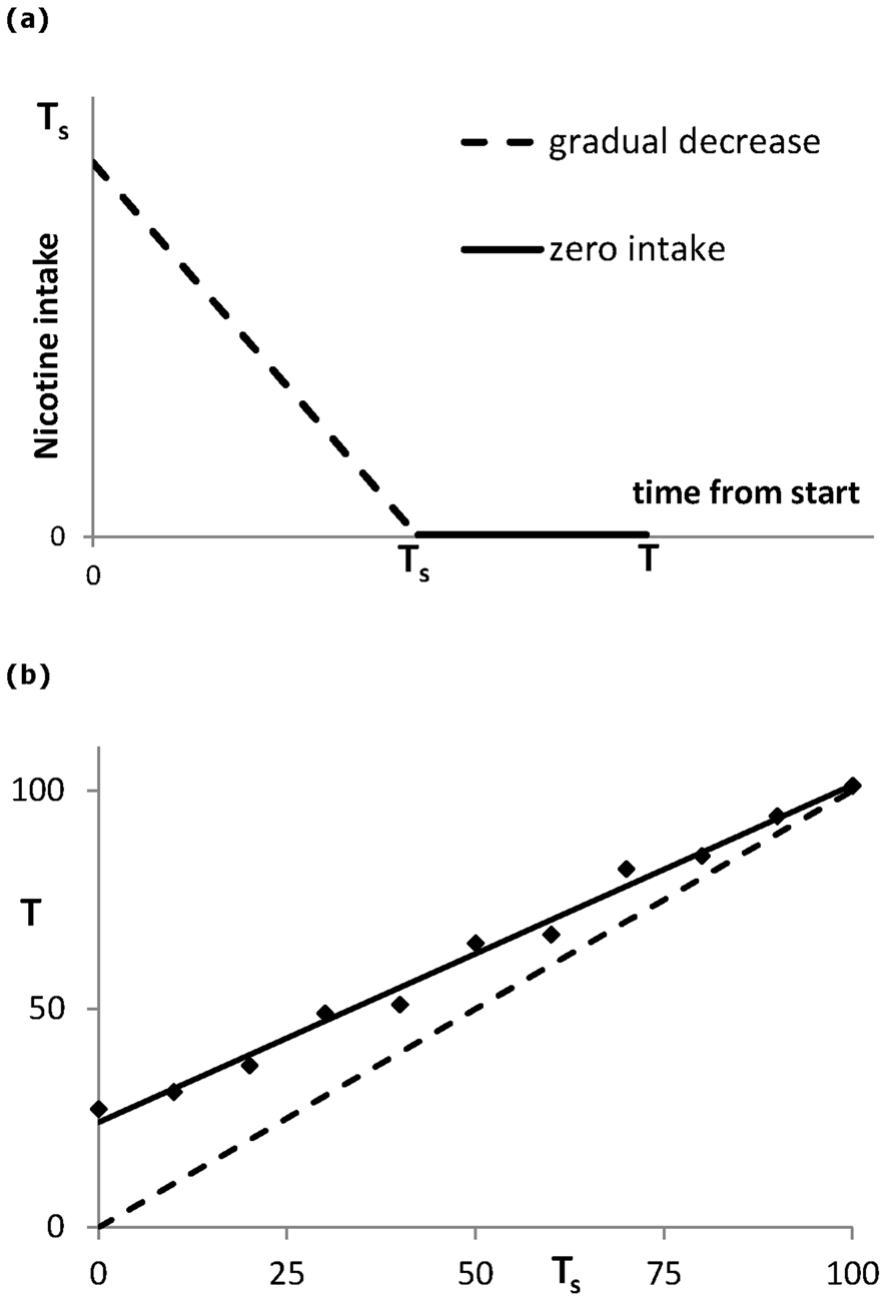
Mixed therapy at which in the first interval [0, *T_s_*] the intake rate is gradually decreased to zero in a linear way. It is followed by the interval [*T_s_*, *T*] in which the nicotine intake is kept zero. The value of *T_s_* varies from 0 to 100 days connecting the two types of therapy analysed before: abrupt cessation versus gradual cessation. (a) Cessation scheme. (b) Results obtained from Monte Carlo simulation of 100 runs at each value of *T_s_*. The variable *T* is the first time of arriving at a score of 30 (or more) of having a negligible nicotine intake during one year after completing the therapy.

Since the rate coefficients *f* and *r* are about a factor 100 larger than *b* and *v*, the state variable *N* will change rapidly when the system starts in some arbitrary initial state. The system will tend to a quasi-steady state, see [11] for more on the analysis of systems of differential equations like (2ab). In the phase plane this quasi-steady state is represented by the function.
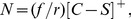
(3)which is found by putting d*N*/d*t* = 0 in Eq.(2a); the corresponding line is called a nullcline, see Figure 2d about the way this nullcline is approached. Trajectories cross this nullcline horizontally. The other nullcline is found from Eq.(2b) by putting d*C*/d*t* = 0. Trajectories of the system traverse this curve in vertical direction on their way to the quasi-steady state being very close to the nullcline given by (3). Let the control have the value *S* = 0.05. In the slow time scale of Eq.(2b) with the constraint (3) the system tends to one of the two stable equilibria of no smoking or steady smoking depending on the starting point in the state plane, see Figure 2a. For a larger control, e.g. *S* = 0.1, the only stable state is the one of no smoking (Fig. 2b), while a negative control, e.g. *S* = −0.03 will definitively lead to a state of steady smoking (Fig.2c). In Figure 3 the equilibrium states are depicted as a function of *S*.

The essential element in our modelling of transitions of smoking behaviour is the presence of two or three equilibria for (2ab) which only may occur in nonlinear systems. Two of the equilibria may get unstable when the parameter *S* changes its value. Furthermore, for values of *S* at the interval [0, 0.085] both these equilibria are stable (bi-stability), see Figure 3. In Figure 1, we presented two choices of model parameter values of (1): (*f*, *g*) = (0.68, 0) and (*f*, *g*) = (0.6, 0.013). Both cases lead to the same bi-stability interval of *S* as we presented in Figure 3. This property holds for a wide range of values of these parameters. Thus, the parameters *f* and *g* are interchangeable for the goal we use them, so that we may as well set *g* = 0.

## Results

### 1. The Effect of a Varying Control

If *S* varies in time, switches from one stable state to the other are possible. For *S* = 0.05 (Fig.2a) a person in the smoking state will move to the non-smoking state if *S* takes a higher value (Fig. 2b). If *S* decreases again the individual may return to the smoking equilibrium if *S* gets a negative value. In order to study this process in time we choose a standard Brownian motion process for the control. This is achieved by taking an Ornstein-Uhlenbeck process having a drift term that sends the control to a deterministic equilibrium *S*_0_ and a diffusion term generating a random change in *S* with expected value 0 and variance ε^2^ *dt* over a time interval *dt*. For that purpose we introduce the standard Wiener process *W*(*t*) being a Brownian motion without drift and with a variance that corresponds with ε = 1. Now we are dealing with a set of stochastic differential equations to which the Itô calculus applies:
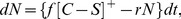
(4a)

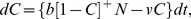
(4b)

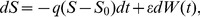
(4c)see [12]. The parameter ε determines the time scale at which the control fluctuates. Attitude changes acting upon the control are assumed to take place in months: ε = 1/(30.3×16) = 2.06×10^−3^. The distribution of realizations of *S* at different times in the stationary state is given in [12]:



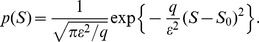



Because of this form of the stationary distribution we replace the parameter *q* by
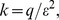
so that the amplitude (st. dev.) of *S* is in the order of 1/*k*. Shifts from smoking to non-smoking and vice versa are expected near *S* = 0.05 with an amplitude slightly exceeding the width of the *S*-interval with the two stable equilibria, see Figure 3. In Figure 4 a realization is given for *k* = 75 and *S* = 0.05. This realisation of the process (4) is approximated by using a forward Euler scheme for the numerical integration of this set of stochastic differential equations [13]. In Figure 5 such a realisation is given for a time interval of 1000 days. The above variation in control leads to a series of sudden changes in smoking behaviour.

### 2. Cessation Strategies

There is a huge literature on cessation strategies. Gradual cessation and abrupt cessation strategies are compared in [14] and [15]. These cessation strategies can be evaluated using model (4abc). We concentrate on the range of values *S*_0_ for which we may expect a successful treatment. From Figures 2 and 3 we conclude that this is the case near *S*_0_ = 0.05, because then the system is bi-stable. A person being a steady smoker may be transformed into a steady non-smoker. At an earlier stage he became a smoker due to low *S*_0_-values (Fig. 2c), e.g. during adolescence.

We assume that at the start of the therapy (*t* = 0) the system is in the stable deterministic equilibrium (*N*, *C*) = (0.68, 0.435) holding for *S*_0_ = 0.05 with an average nicotine intake of 0.262[mg/h]. For *t* >0 the control fluctuates as described in Section 3 with the parameters ε and *k* having the same values as given there. The abrupt cessation therapy taking place during the time interval (0, *T*) is set up as follows: the control is increased and set at *S*_0_ = 0.1 and there is no nicotine intake at all (*f* = 0). At the end of the time interval *S*_0_ and *f* are reset at their original values. During the therapy the variable *C* decays as described in Eq.(2b) with *f* = 0. The success of the therapy strongly depends on the level of *C* at the end of the therapy. We carried out a Monte Carlo simulation running the system 100 times. This simulation is repeated for different values of *T*. In Figure 5a it is seen how many cases are successful; meaning that during one year after the end of the therapy they have stayed below 5% of the nicotine intake they had before the therapy. The success rate stabilizes after 30 days reaching a score of about 30%, which agrees reasonably well with the one year abstinence rates reported in the literature on therapy supported cessation [16]–[19] (much lower success rates, as low as 3%, are reported without some form of external enforcement of the cessation [20]). In case of a gradual cessation the intake of *Q* decreases linearly from 0.262 to 0 over the interval (0, *T*), we see in Figure 5b that it now takes about 100 days to reach this success rate.

The two types of treatment each have their (dis)advantages: the difference in the required length of the therapy is quite large: the gradual cessation needs much more time. Thus, abrupt cessation seems to be the best strategy [21]–[22]. However, gradual cessation may ease the transition to a new daily life without nicotine. Therefore it is worthwhile to analyse an intermediate type of therapy in which one starts with an interval of gradual cessation followed at *t* = *T_s_* by an interval of full abstinence, see Figure 6a. Such treatments have been studied in [19]. We test this with Monte Carlo simulations as before. At each value of *T_s_* we consider therapies of length *T* = *T_s_*, *T_s_* +1, …., for which we carry out 100 runs. In Figure 6b, we present the first time *T* (in days) the success rate equals or exceeds 0.30 for the different lengths of the interval of gradual cessation. For instance, for a gradual phase of *T_s_* = 21 days, complete abstinence should be maintained for at least 18 days. Indeed, in [19] it is reported that active enforcement of abstinence for a sufficient amount of time after cessation leads to much lower 1 year abstinence rates. From our set of mixed therapies one may determine the minimum active enforcement duration necessary after the start of the full cessation time *T_s_*. One may as well wish to select the appropriate scheme given the total length *T* of the therapy. From the regression line given in Figure 6b the required corresponding value of *T_s_* can be read off.

## Discussion

From a set of basic assumptions on nicotine intake, (self)control and craving we derived a dynamical model for smoking consisting of a set of two coupled nonlinear differential equations. For the chosen values of the model parameters three dynamical regimes may occur depending on the control parameter *S*: no smoking, steady smoking and a bistable state in which both types of behaviour may occur. We also considered the case that the control *S* depends on time. We have chosen a Brownian motion process for this change in *S*. In this way we introduced noise leading to a scenario in which a steady smoker may stop smoking for some time when the control takes high positive values, and may restart, if *S* takes negative values. This relapse does not depend only upon the control but also on the actual level of craving. At the end of a therapeutic treatment craving must be sufficiently low. The present model makes it possible to quantify the risk that this craving level does not meet the requirements for having a successful treatment. Moreover, from simulations it is concluded that next to abrupt and gradual cessation therapies it is worth to consider a mixed strategy starting with a phase of gradual decrease of nicotine intake followed by a phase of no intake at all. In this way residual craving at the end of the treatment can be put at a safe low level. Furthermore, the therapist and client may choose a duration of the therapy that fits them the best: for a duration *T* of the therapy at the interval [30, 100] the regression line in Figure 6b yields the optimal moment *T_s_* of ending the gradual decrease of nicotine intake and starting the stage of zero intake. For having the best result from the cessation therapy, the craving decay parameter *v* should be estimated as accurate as possible. Its reciprocal is an indication for the time craving is still felt. We put it at 26 days. In the literature values starting from 10 upto over 31 days are found [23]. It is indeed expected that from person to person the parameter *v* may differ considerably. If from surveys a significant correlation can be found between the system parameters and traits that come with drug addiction, a more accurate parameter estimate can be made using information obtained at the intake of the client, so that the length of the therapy can be adjusted. Studies are already made on personalized treatments based on pharmacotherapy taking in account the composition of gene variants acting upon nicotine related neural pathways [24]. It is remarked that administering medicines, such as bupropion, require much more care than just changing the length of the treatment.

In our modelling of the interaction between nicotine intake and craving we made a number of assumptions. Nonlinear functional relations are essential in grasping the essentials of the process. In our model the first term in the right hand side of both equations (2a) and (2b) are responsible for the possibility of having a bi-stable system. Under a fluctuating control the phenomenon of repeatedly resuming smoking shows up in a realistic way in the simulation (Fig.4). Therefore, there is no need to search for other nonlinear mechanisms. Especially, as detailed information on some functional relations are missing, we preferred to keep it simple and assumed that the choice of linearity would suffice. This particularly applies to the suppositions that the nicotine intake depends linearly upon the craving for C - S >0 (Fig.1a) and that the level of craving equals the fraction of activated NARs. At the other hand the model in its present form still explains a nonlinearity as it is found in [25], where it is concluded that cigarette smoking saturates the level of turned on NARs. In our model we recover this property in the equilibrium state of Eq.(2ab) with *C = Q*/(*Q* + *vr*/*b*), where *C* is the level of craving and *Q* the nicotine intake. This relation represents a Michaelis-Menten type of kinetics [11].

The present model only applies to the intake of nicotine and does not help to qualify existing therapies on other elements of the treatment, such as offering substituting goodies or increasing the awareness of the risks of smoking. These, of course, do play a role in a successful completion of a treatment and in the risk of a restart. Administration of nicotine in other ways than by regular cigarettes needs in our model to be included in the total nicotine intake and is only allowed in the phase of gradual decrease.

The model may be extended in several directions, in order to account for other empirical phenomena such as, social influences on cessation success [3], [26], hereditary aspects of nicotine addiction [27]–[28], and withdrawal [30]. These extensions may result in additional equations or modifications of the current system of equations. It is expected that the qualitative dynamics as present in the current system will be preserved.

## Supporting Information

Appendix S1 **Stationary and non-stationary Poisson processes**
(DOCX)
