# Supplementary material for: Transitions in Smoking Behaviour and the Design of Cessation Schemes
Source: PLoS One. 2012 Oct 11;7(10):e47139. doi: 10.1371/journal.pone.0047139 (PMC3469545; doi:10.1371/journal.pone.0047139)
Supplement: Appendix S1 — Stationary and non-stationary Poisson processes (DOCX) [file pone.0047139.s001.docx]

**Appendix S1 Stationary and non-stationary Poisson processes**

With a Poisson process one may describe the occurrence in time of stochastic events having a given mean frequency of λ events per unit of time. Within a time interval (*t*, *t* + Δ*t*) an event takes place with a probability that is approximated by λ Δ*t* for Δ*t* sufficiently small. Events up to time *t* do not play any role. Let from some given time *t*_0_ we consider the time interval that ends with the first time *t*_1_ that an event takes place. The Poisson process has the property that the length of that time interval has an exponential distribution with the same parameter λ. The probability that the length of the interval is between *L* and *L* + d*L* satisfies λexp(-λ*L*)d*L*. Allowing that the interval at time *t*_0_ starts with an event we conclude that the time between two subsequent events is also exponentially distributed having an expected value 1/λ:

$E\left[ L \right]=\int_{0}^{\infty} L\lambda exp(-\lambda L)dL=1/\lambda$. (A1)

For a non-stationary Poisson process with

$\lambda\left( L \right)=\alpha L$

we obtain for the expected value of the length of the interval between two events

$E\left[ L \right]=\int_{0}^{\infty} L\lambda(L)exp\{-\int_{0}^{L} \lambda\left( l \right)dl\}dL=\sqrt{\frac{\pi}{2\alpha}}$. (A2)
